# *Erysipelothrix* Spp.: Past, Present, and Future Directions in Vaccine Research

**DOI:** 10.3389/fvets.2020.00174

**Published:** 2020-04-15

**Authors:** Tanja Opriessnig, Taya Forde, Yoshihiro Shimoji

**Affiliations:** ^1^The Roslin Institute and The Royal (Dick) School of Veterinary Studies, University of Edinburgh, Midlothian, United Kingdom; ^2^Department of Veterinary Diagnostic and Production Animal Medicine, College of Veterinary Medicine, Iowa State University, Ames, IA, United States; ^3^Institute of Biodiversity, Animal Health & Comparative Medicine, University of Glasgow, Glasgow, United Kingdom; ^4^National Institute of Animal Health, National Agriculture and Food Research Organization, Tsukuba, Japan; ^5^Research Institute for Biomedical Sciences, Tokyo University of Science, Chiba, Japan

**Keywords:** *Erysipelothrix* spp., history, immune protection, vaccines, review

## Abstract

*Erysipelothrix* spp. comprise a group of small Gram-positive bacteria that can infect a variety of hosts including mammals, fish, birds, reptiles and insects. Among the eight *Erysipelothrix* species that have been described to date, only *Erysipelothrix rhusiopathiae* plays a major role in farmed livestock where it is the causative agent of erysipelas. *E. rhusiopathiae* also has zoonotic potential and can cause erysipeloid in humans with a clear occupational link to meat and fish industries. While there are 28 known *Erysipelothrix* serovars, over 80% of identified isolates belong to serovars 1 or 2. Vaccines to protect pigs against *E. rhusiopathiae* first became available in 1883 as a response to an epizootic of swine erysipelas in southern France. The overall vaccine repertoire was notably enlarged between the 1940s and 1960s following major outbreaks of swine erysipelas in the Midwest USA and has changed little since. Traditionally, *E. rhusiopathiae* serovar 1a or 2 isolates were inactivated (bacterins) or attenuated and these types of vaccines are still used today on a global basis. *E. rhusiopathiae* vaccines are most commonly used in pigs, poultry, and sheep where the bacterium can cause considerable economic losses. In addition, erysipelas vaccination is also utilized in selected vulnerable susceptible populations, such as marine mammals in aquariums, which are commonly vaccinated at regular intervals. While commercially produced erysipelas vaccines appear to provide good protection against clinical disease, in recent years there has been an increase in perceived vaccine failures in farmed animals, especially in organic outdoor operations. Moreover, clinical erysipelas outbreaks have been reported in animal populations not previously considered at risk. This has raised concerns over a possible lack of vaccine protection across various production species. This review focuses on summarizing the history and the present status of *E. rhusiopathiae* vaccines, the current knowledge on protection including surface antigens, and also provides an outlook into future directions for vaccine development.

## Introduction

Erysipelas in animals and erysipeloid in people are both caused by infection with the Gram positive bacteria *Erysipelothrix* spp. which belong to the family *Erysipelothrichaceae*, order *Erysipelotrichales*, class *Erysipelotrichia* and phylum *Firmicutes* (1). *Erysipelothrix* spp. can be divided into eight different species: *Erysipelothrix rhusiopathiae* (2, 3), *Erysipelothrix tonsillarum* (4), *Erysipelothrix* species 1, *Erysipelothrix* species 2, and *Erysipelothrix* species 3 (5), *Erysipelothrix inopinata* (6), *Erysipelothrix larvae* (7) and *Erysipelothrix piscisicarius* sp. nov. (8). Isolates associated with these species were identified in mammals, birds and fish (*E. rhusiopathiae, E. tonsillarum* and *E. species* 1, 2, and 3; *E. piscisicarius*), a vegetable peptone broth (*E. inopinata*) or insects (*E. larvae*). *Erysipelothrix* spp. have a worldwide distribution and are considered ubiquitous with most identified isolates belonging to *E. rhusiopathiae* (9). The most important reservoir for *E. rhusiopathiae* are pigs, where an estimated 30-50% of healthy pigs appear to harbor the organisms in tonsils or lymphoid tissues (10).

Erysipelas describes an acute bacterial disease in pigs and other species, often characterized by raised, red skin patches (11). Not surprisingly, the term “erysipelas” is derived from the ancient Greek “ερ*υσ*íπ*ελας*” which means “rose” or “red skin” (12, 13). While skin discoloration in pigs may occur due to various etiologies and the term “swine erysipelas” is not very specific, it has still become a synonym for *Erysipelothrix* spp. infection in pigs and is well-known by veterinarians and other occupations connected to the food animal industry (14). Today, erysipelas is also used to describe clinical manifestations associated with this bacterium in other species including mammals, fish, birds and reptiles (15, 16). In humans, where the disease was first described in 1870 (17) since 1909 the term “erysipeloid” is used (18).

**Figure 1:**
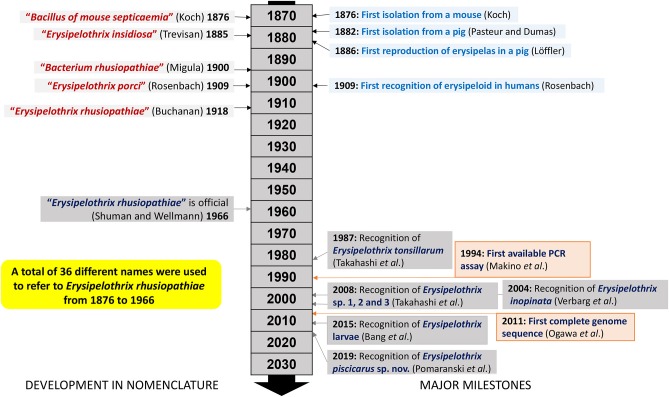
Timeline and milestones in *Erysipelothrix* spp. nomenclature and research.

Prior to successful bacterial isolation and characterization, erysipelas was thought to be anthrax of swine as it resembled some of the clinical manifestations in cattle (9). During 1877–1878 the “great swine plague” (i.e., classical swine fever) moved through the United States, England and Scandinavian countries before entering and spreading through mainland Europe (14). Losses associated with these epidemics likely prompted early bacteriologists to investigate swine diseases (14). In 1876, Robert Koch successfully isolated a bacterium from a mouse inoculated with putrefying blood (19). Koch initially designated the bacterium “*Bacillus of mouse septicemia*” (19). Since Koch's discovery, the bacterium changed its name a total of 36 times; *Erysipelothrix rhusiopathiae* was officially designated as its scientific name only in 1966 (20) and has been in use ever since (Figure 1). This specific name was created from the Greek words “erysipelas” (rose, red skin), “trix” (hair), “rhusius” (reddening), and “pathus” (disease). During a swine erysipelas outbreak in southern France from 1882-1883 Louis Thuillier successfully isolated the bacterium from pigs with “rouget” as erysipelas is called in France (21). At approximately the same time Friedrich Löffler also isolated the bacterium but only published his findings after completing the first accurate description of *E. rhusiopathiae* and reproducing erysipelas in experimentally infected pigs in 1886 (22) (Figure 1).

**Figure 2:**
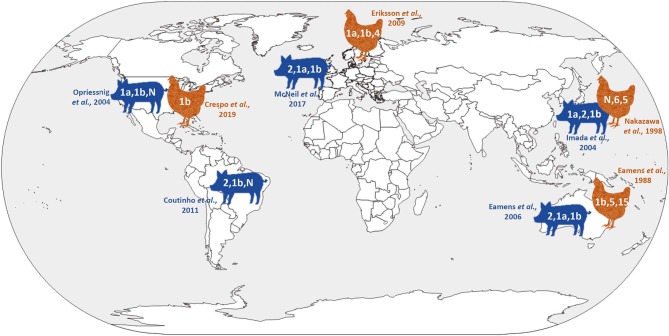
Current knowledge on global *Erysipelothrix* spp. serovar distribution in pigs and poultry.

Historically, *Erysipelothrix* isolates have been divided into at least 28 serovars based on specific polysaccharide complexes that can be demonstrated by double agar-gel precipitation testing using type specific rabbit antiserum (23, 24). Initially there were several serotyping systems, and the most commonly used recognized two serovars, A and B, with a third group (N) for all isolates that did not react with A or B antiserum (25). Because this typing scheme quickly became unpractical with more and more isolates falling in the N group, a new system was created which is used to this day (23, 24). In this system, the serovars are indicated by consecutive Arabic numbers in order of discovery (i.e., 1–26), serovar 1 and sometimes 2 may be further subdivided and indicated by small letters (i.e., 1a, 1b, 2a, 2b). The old A and B serovars correspond to 1 and 2 in the new system. Serovar N, which lacks serovar-specific antigens and is still used in the new serotyping system, has been shown to arise from various other serovars following genetic mutation (26, 27). Certain serovars have historically been associated with particular *Erysipelothrix* species including serovars 1a, 1b, 2, 4–6, 8, 9, 11, 12, 15–17, 19, 21, 23, and N with *E. rhusiopathiae*, serovars 3, 7, 10, 14, 20, 22, and 24–26 with *E. tonsillarum*, serovar 13 with *E. species* 1 and serovar 18 with *E. species* 2 (28). However, this is not absolute and certain serovars can be associated with more than one species (5, 29). Serotyping studies have indicated that, with some geographic differences, serovars 1a, 1b, and 2 are most widely distributed in pigs and likely of greatest importance overall (24, 29–36) (Figure 2). Serotyping is not performed routinely, and serovars are not species specific or phylogenetically informative (37). It has been shown that the chromosomal locus responsible for determining antigenicity of serovars 1a and 2 is involved in the virulence (26); however, the overall importance of serovar in pathogenesis and immune protection is largely unknown.

Erysipelas vaccines are commonly used in pigs (38). In this species, most breeding herds in almost all pork producing regions are regularly vaccinated (Source: https://www.nadis.org.uk/disease-a-z/pigs/erysipelas/). In contrast, growing pigs are not commonly vaccinated against erysipelas as they are expected to have passively acquired antibodies when leaving the breeding farm. However, if there is a perceived high erysipelas pressure in grow-finish farms, growing pigs are also vaccinated (Source: https://www.nadis.org.uk/disease-a-z/pigs/erysipelas/). Erysipelas vaccines are less commonly used in poultry (e.g., turkeys or laying hens) (39) or sheep (40). Turkeys used for meat production can be vaccinated but this may be labor intensive (39). Breeder turkeys are vaccinated four weeks before onset of egg production (39). Chickens (almost exclusively layers) are vaccinated at least twice (or more often) 2–4 weeks apart (35). Ewes are not regularly vaccinated outside the large sheep production regions of Australia/New Zealand due to a low cost-benefit ratio of vaccination. However, to prevent erysipelas arthritis in lambs, vaccinating ewes prior to lambing will provide immunity in lambs for up to 8 weeks (Source: https://www.zoetis.com.au/_locale-assets/faq/erysipelas.pdf).

While for many decades *Erysipelothrix* spp. infections had minimal impacts on livestock production, erysipelas appears to be re-emerging today due to changing environmental conditions, changes in welfare regulations, an increase in outdoor organic farming (41), and changes in antimicrobial usage, with global reductions and eventual bans anticipated in the very near future (42). Sporadic outbreaks are being reported in various settings such as in farmed animals (31, 35, 43–46) and more specifically organic farms (47, 48), in fish (16, 49) as well as globally in wild animal species (50, 51). Climate change could favor increased bacterial loads and persistent environmental contamination. Alternatively, erysipelas outbreaks may be associated with changes in host resistance. Further explanations for re-emergence of *E. rhusiopathiae* in livestock could include decreasing vaccine efficacies, adaptations in the *Erysipelothrix* spp. populations allowing certain isolates to better survive in vaccinated populations or both. With the ever-expanding knowledge on erysipelas in different animal species and the perceived increase of clinical cases in livestock and wild animal species in recent years, it appears important to revisit the accumulated background information on protection and vaccination against *Erysipelothrix* spp. and gain perspectives for the future.

## Disease Manifestation

Erysipelas is known to cause three main clinical manifestations in animals: acute, subacute, and a chronic disease (38). In addition, there is also subclinical disease. For pigs, acute disease is characterized by a sudden onset of clinical signs and can include acute death, fever, withdrawal from the herd, squealing, stiff stilted gait, weight shifting, depression, inappetence, diamond skin lesions which may appear 2–3 days after infection and disappear 4–7 days after first appearance, and necrotic lesions on tail, ears, and posterior aspect of the thighs. As a milder variation of the acute form, the subacute disease manifestation has similar but less severe clinical signs with fewer skin lesions; overall the subacute form may remain unnoticed, i.e., subclinical (38). The chronic manifestation of erysipelas, which may follow acute or subacute disease in pigs, is most commonly characterized by signs of arthritis (stiffness, enlargement) as early as three weeks after initial infection, and/or signs of cardiac insufficiency, sometimes with sudden death (38). In breeding sows, abortions or increased pre- and postpartal vulval discharge may be observed, as well as smaller litter sizes and reduced numbers of live born piglets (52). The chronic form of erysipelas may also affect growth rate and increase losses of cuts in meat packing plants (53).

Erysipelas can occur in a wide range of farmed poultry including turkeys, broiler chickens, laying hens, geese, pheasants and quails. Layers may simply suffer from sudden death due to acute septicaemia. Normally few birds may be depressed initially with mortality starting within 24 h. There may also be dramatic decrease of egg production (35) and conjunctival edema (46). Unsteady locomotion and lack of coordinated movement have also been reported (13). The disease can be fatal in young adult turkeys and ducks, with affected birds developing severe hemorrhages in breast and leg muscles (54). Turkeys with vegetative endocarditis usually do not have clinical signs and may die suddenly (13). Outbreaks have been reported in 2–3 day old poults (55) or commercial breeder flocks of quails (56). In sheep, the most common clinical manifestation is polyarthritis, typically presenting in 2- to 6-month-old lambs (57). Chronic ovine erysipelas is often restricted to joints with subchondral bone involvement (57). While slaughter condemnations in farmed animals other than pigs rarely occur, chronic cases that reach the slaughterhouse may pose a zoonotic risk to workers and infection of slaughterhouse employees has been reported (56).

## Economic Impact

*Erysipelothrix rhusiopathiae* is of economic importance in pigs, poultry and lambs where outbreaks can cause high losses. Today the majority of pig breeding herds in Europe, North America, and South America are routinely vaccinated (every 4–6 months) against erysipelas, and erysipelas vaccine usage is increasing in poultry. From October 2018 to September 2019, ~10,683,595 doses of autogenous *E. rhusiopathiae* vaccines, 72,440,500 doses of attenuated vaccines and 33,257,460 doses of bacterins were released in the USA across species (Source: USDA, Dr. Paul Hauer, 2019). In the UK, during 2018, ~1,359,120 doses of inactivated vaccines including bivalent and trivalent products were sold (Source: Kynetec, UK data, 2019). In pigs, large scale outbreaks are an ongoing problem in some areas including the USA, where documented outbreaks occurred in 1989–1990 (58) and again in 2000–2001 (32). An outbreak is often devastating for affected producers, and for organic chicken productions in particular, where it may lead to permanent closure of the farm. Additionally, subclinical disease in pigs can be of great importance when it leads to abattoir condemnations, as at this point the producers have already incurred the maximal possible cost on the individual pigs. In modern food animal production, the precise economic impact of *Erysipelothrix* spp. is often multifaceted and likely underestimated.

**Figure 3:**
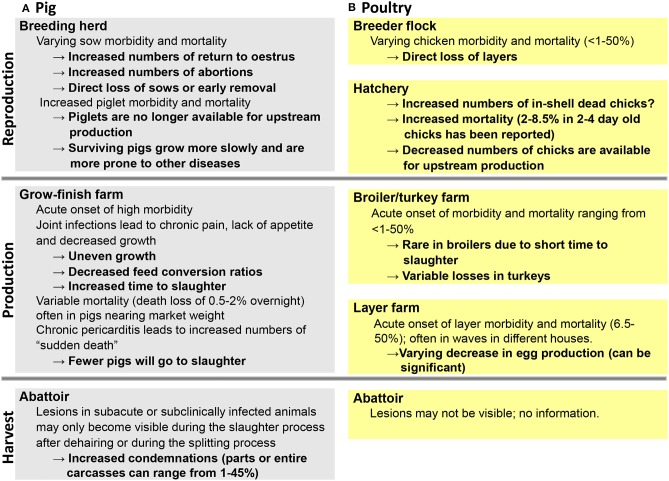
Impact of erysipelas on different production stages in pigs **(A)** and poultry **(B)**.

Economic losses can be directly associated with high morbidity and mortality rates during an acute outbreak (Figure 3) resulting in a reduction of animals on a given farm (43, 45), including significant egg drop in layers (47). In addition, as a result of joint pain and lameness due to the chronic form of the disease, there may be substantial decreased weigh gain, subsequently resulting in an increased time to slaughter (57). Finally, there may be increased numbers of condemned carcasses at slaughter due to lesions that may only become visible during scaling and dehairing processes or at the splitting floor (53, 59). In such cases, producers may be completely unaware of any ongoing erysipelas infection on the farm (Figure 3). When 153 arthritic joints from Canadian slaughter pigs were examined for bacteria, *E. rhusiopathiae* was found most frequently and identified in 45% of the samples (60). However, based on meat inspection rules in the European Union enforced in January 2006, if cutaneous erysipelas is detected prior to slaughter on farm, slaughtering of affected pigs must be deferred for at least 15 days from disappearance of typical lesions to guarantee meat safety (61).

Overall, it is often difficult to get estimates of the direct impact of erysipelas on a population, as *E. rhusiopathiae* infections are not reportable to health authorities in most countries. In Japan, where swine erysipelas is a reportable disease, ~2,000 pigs are affected annually, which is equivalent to 0.0125% of finishing pigs produced in the country. PCR assays have been developed for improved detection of contaminated pigs at Japanese slaughterhouse (62). Once the infection occurs in farms, pigs often become carriers of the organism and disperse the organism in their feces, resulting in contamination of their environment. Since the disease may also cause abortions in breeding herds, the total economic losses may become intolerable for affected farms. Analysis of erysipelas case trends are rarely published from other countries. However, a recent analysis of epizootic swine erysipelas cases from 2006 to 2017 in the Ukraine found a total of 39,952 cases during this time period, mainly in the Southeast region of the country (63).

Economic losses can be assessed via two routes: (I) by using production and economic models to value the production losses due to disease (presently not available for erysipelas) and (II) by partial budgeting approaches with “rules-of-thumb.” An example of the latter has been published at https://thepigsite.com/articles/erysipelas-why-is-it-still-a-problem-after-100-years (64). Based on the provided example calculations for the United Kingdom, a 400 sow breeding herd with an acute outbreak could lose 388 pigs due to abortions and sow mortality. For 2008, this would have cost GBP 14000. A late/chronic erysipelas outbreak in a 600 head finisher herd could result in ~58 pigs being euthanized and 31 condemned for a total loss of GBP 17451 over a six-month period (64). In a recent study focusing on global trends in infectious diseases in pigs, pathogens were ranked based on overall publication counts from 1966 to 2016 (65). The top 40 pathogens included 16 viruses, 15 bacteria, eight helminth parasites and one protozoan, with *E. rhusiopathiae* ranking 30th. This relatively low representation in the scientific literature may reflect an overall neglect of investigating long-standing endemic bacterial pathogens.

## History of Erysipelas Prevention Methods

### Attenuated Vaccines

**Figure 4:**
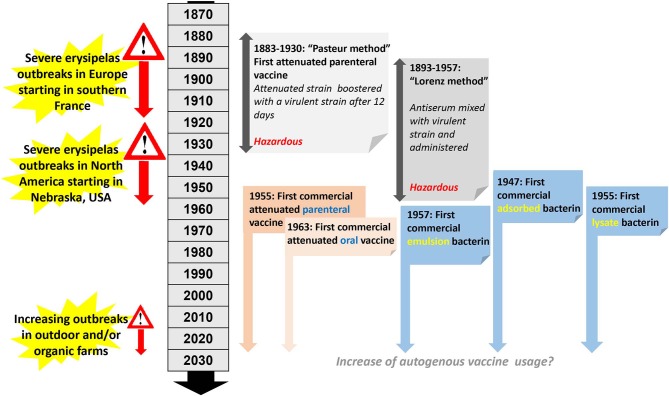
Timeline and milestones of *E. rhusiopathiae* vaccine development in pigs.

The development of bacterial vaccines for pigs began at the end of the nineteenth century (Figure 4) when the very first erysipelas vaccine became available (66). This initial attempt to produce an attenuated erysipelas vaccine was in direct response to a major swine erysipelas outbreak in southern France (67). After successful isolation of *E. rhusiopathiae* from affected pigs, the virulence of the bacterium was enhanced by passage through pigeons. This was followed by passages through rabbits which are only weakly susceptible to *E. rhusiopathiae* infection. Interestingly, this resulted in an increase of virulence of the isolate for rabbits and a decrease of virulence for swine (67). The so derived attenuated vaccine was used in pigs by injecting the attenuated culture, followed by another injection 12 days later using the virulent culture (Figure 4). This method was used from 1886 to 1892 in more than 100,000 pigs in France (68), and was subsequently adapted in other European countries (but never in North America), and used for pig vaccinations until 1930 (69). As this method is hazardous, it has since been abandoned. Subsequently, live-culture erysipelas vaccines for parenteral use in pigs have been available since 1955. The first vaccine of this type was named erysipelas vaccine avirulent or EVA (70). For this particular product, large volumes of the bacteria were injected into mice, pigeons, and pigs without resulting in any clinical signs, hence the vaccine was considered avirulent (70). Oral live erysipelas pig vaccines were developed during the 1960s (71, 72). The idea of orally vaccinating pigs originated by observations made from mass vaccination against human polio, and initial studies using experimentally vaccinated and challenged pigs indicated efficacies of 85–90% (71). Methods for attenuation included air-drying (73), passage in media containing acridine dyes (74), or passage through rabbits or chicken embryos (75). Specifically, oral vaccines were found to be safe in pigs without generating carriers or shedders (76) and vaccines were found to be stable both in the dried and liquid stage (72). Subsequently, swine oral vaccines were also examined for use in turkeys and were considered potentially useful (77). Attenuated vaccines are still in use today, administered via drinking water, intramuscular injection or even air exposure and are useful for vaccinating larger animal populations housed indoors including poultry and pigs. It is recommended to discontinue antibiotic treatment 8–10 days before vaccination (75). A single dose of an attenuated vaccine is sufficient to provide protection which varies across products but in general is guaranteed for up to 6 months. Usage of any attenuated vaccine strain is always associated with certain risks. While reversion to virulence of attenuated vaccines has not been reported in Western countries until now, in Japan attenuated erysipelas vaccines have been associated with chronic outbreaks of erysipelas (78).

### Serum-Culture Co-immunization

In 1891, Emmerich and Mastbaum discovered that when rabbits where hyperimmunized against swine erysipelas, their serum had curative properties (79). In 1893 a simultaneous serum-culture intervention was introduced for immunization, which consisted of concurrently injecting viable culture and a hyperimmune serum (79). This principle evolved into two methods. In the “Leclainche method,” a virulent culture was mixed with antiserum just before inoculation. In contrast, in the “Lorenz method,” named after a German veterinarian and widely used in Germany for more than 50 years (80, 81), antiserum and virulent culture were given at the same time but at different inoculation sites (Figure 4). The Lorenz method was introduced to Nebraska, USA in 1938 and by 1953, 26 U.S states were using this method. Similar to the Pasteur vaccine, ultimately these methods were considered to be hazardous; after other active immunization protocols became available, the usage of these methods eventually ceased (79).

### Inactivated Vaccines

The first large-scale swine erysipelas outbreaks in the USA occurred in South Dakota, USA between 1928 and 1930 (82, 83). As a direct consequence, during the late 1940s, shortly after World War II, the first inactivated erysipelas vaccines for usage in pigs were developed (84, 85). Specifically, usage of a bacterin, consisting of formalin-killed whole *E. rhusiopathiae* culture adsorbed on an aluminum hydroxide gel was first reported in 1947 in East Germany (85) and licensed in the US in 1953 (Figure 4). Bacterins often use selected serovar 2 isolates that produce a soluble immunogenic product when grown in a complex liquid medium containing serum (75). This substance has been described as a glyco-lipoprotein (86) and most of it is released into the medium and is considered the necessary immunogenic ingredient for bacterins (75). Further characterization of the glyco-lipoprotein fraction during the 1990s revealed a 64–66 kDa protein fraction (87, 88) later described as *s*urface *p*rotective *a*ntigen (Spa) A (89, 90). The main downside of the original bacterin was a protective immunity which only lasted 2-4 months. In an attempt to improve the formalin-killed Traub adsorbate bacterin, an emulsion bacterin was prepared using a water-in-oil emulsion (manide mono-oleate and a light mineral oil; bayol F) (91, 92). Pigs vaccinated with this emulsion bacterin developed immunity lasting at least 7–8 months or 237 days (91, 92). Bacterins developed at this time were also shown to be effective in turkeys (69, 93). A modified bacterin version, the lysate bacterin was developed in the Institut Merieux, Lyon, France in 1953 (94), licensed in the USA in 1955 (Rhusigen, Allied Laboratories, Inc., Indianapolis), and is similar to a regular bacterin with the exception of lysis of bacterial cells. Anecdotal evidence suggested that the bacterin could also be used with anti-swine serum to achieve immediate protection (94). Bacterins are given by subcutaneous or intramuscular routes and a booster vaccination may be required for some products, which typically needs to be administered 2–3 weeks after first vaccination. Manufactured inactivated vaccines have to follow strict quality controls and each batch needs to be tested for relative strength. Early on, this required extensive animal experiments in laboratory mice and pigs for the determination of efficacy (80). The discovery of skin scarification, allowing testing of more than one culture in production on a single animal, was considered a major improvement (95). The World Health Organization (WHO) established International Standards for erysipelas vaccines and antisera concerning potency testing in mice (80). Today, potency testing in Europe is generally performed with enzyme-linked immunosorbent assays (ELISAs), which have largely replaced animal experiments (96, 97). This change has reduced animal usage by over 80% (98, 99).

### Passive Immunization

Passive immunity is a way to immediately but temporarily protect an animal for ~2 weeks by administering a commercially available antiserum. This treatment utilizing hyperimmune serum, usually obtained from horses, was introduced in 1899, several years after it had been developed for the serum-culture co-immunization (75). Studies in mice receiving antiserum prepared in rabbits or horses indicated protection even against serologically different isolates (100). Until introduction of antibiotics in pig production in 1949 (101), hyperimmune serum was the only means by which to treat affected pigs (82), but ultimately its usage decreased in later years. In general, the preventive dose is half the therapeutic dose (75). While passive immunization may be useful in protecting pigs during an acute outbreak, it is not very practical as antisera are not readily available and protection is not long-lasting, and therefore this method is not widely used today.

## Currently Available Commercial Vaccines

Today several commercial vaccines to protect pigs, lambs and poultry from the negative impacts of *Erysipelothrix* spp. are available (Table 1). Although they are not very effective in preventing chronic arthritis, its frequency and severity are usually lower in vaccinated pigs compared to non-vaccinated pigs (105). Essentially all commercial vaccines available to date are based on serovar 1a or serovar 2 isolates, where serovar 1a is more commonly used in attenuated vaccines and serovar 2 is more commonly used in bacterins. Since the first introduction of attenuated erysipelas vaccines and bacterins, little has changed in terms of which isolates are being utilized (Table 1). For attenuated vaccines in modern husbandry, care needs to be taken with routine waterline disinfection, anything that may prevent the animals from drinking water (Source: http://www.pig-world.co.uk/features/water-medicating-pigs-what-you-need-to-know.html) and antibiotic administration at vaccination (106), as these may negatively impact vaccine efficacy. For bacterins various adjuvants are added to provide longer immunity. There are notable differences in the geographic distribution of the type of erysipelas vaccines, as live bacterial vaccines are rarely used in Europe (29) while they are common in other swine producing regions including North and South America (32) and Asia (78, 107) (Table 1).

**Table 1:** Basic information on selected commercial *Erysipelothrix rhusiopathiae* vaccines.

**Vaccine^a^**	**Manufacterer**	**Isolate ID**	**Serovar**	**Year of initial strain description**	**Type**	**Availability**	**Species**
ERY VAC FD	ARKO Laboratories, Ltd.	Not disclosed	1a		Attenuated	USA	Turkeys
ERY VAC 100	ARKO Laboratories, Ltd.	Not disclosed	1a		Attenuated	USA	Pigs
Ingelvac^®^ ERY ALC	Boehringer-Ingelheim Vetmedica	R-9	1a	1944 (102)	Attenuated	USA	Pigs
Suvaxyn^®^ E-Oral	Zoetis	31	1a	1963	Attenuated	USA, Canada	Pigs
Swine erysipelas live (seed lot vaccine)	National Veterinary Assay Laboratory, Ministry of Agriculture, Forestry and Fisheries	Koganei 65-0.15	1a	1971 (103)	Attenuated	Japan	Pigs
Ruvax^®^	Boehringer-Ingelheim Vetmedica	Unknown	2	Unknown	Lysate bacterin	EU	Pigs
Parvoruvax^®^	Ceva Animal Health, Ltd				Lysate bacterin	EU (not Malta), Brazil, Caucasus, Mexico, Moldova, Switzerland, Russia, Middle East	Pigs
Coopers^®^ ERYGUARD^®^	Coopers Animal Health (Intervet Australia Pty Ltd/MSD Animal Health Australia)	Unknown	2	Unknown	Bacterin	Australia	Pigs, sheep/lambs
ERYSENG^®^	Hipra	R32/E11	2	1968 (104)	Bacterin	EU, UK, Brazil, Argentina, Mexico, Taiwan, Thailand, Republic of Belarus, Ukraine, Russia, Georgia, Peru	Pigs
MaGESTic^®^ 7	Merck Animal Health	SE-9	2	1948 (84)	Bacterin	USA	Pigs
Nobilis^®^ Erysipelas	MSD Animal Health	M2	2	1946	Bacterin	EU, UK	Turkeys
Porcilis^®^Ery						EU, UK, South Africa	Pigs
ER Bac^®^Plus	Zoetis	CN3342	2	1963	Bacterin	USA	Pigs
Farrowsure Gold						USA, Canada, South Africa	
Eryvac^®^	Zoetis	Unknown		1963 (Seed Source: Medical Research Council, National Institute for Medical Research, London)	Bacterin	Australia, New Zealand	Pigs, sheep/lambs
Suvaxyn^®^ Parvo/E	Zoetis	B-7	2	1989 (Spain)	Bacterin	USA, Canada, EU	Pigs
Swivac ERA	Kyoritsu Seiyaku Corp.	SpaA only	1a	2011	Subunit	Japan	Pigs
SUIMMUGEN^®^rART2/ER	KM Biologics Co., Ltd.	SpaA only	2		Subunit	Japan	Pigs

a *Erysipelas vaccines for usage in pig breeding herds are often available monovalent, or bi- or trivalent in combination with porcine parvovirus and/or Leptospira spp. As monovalent, bivalent or trivalent products from the same company often contain the same Erysipelothrix rhusiopathiae isolate, only one product per isolate is listed*.

In breeding herds, vaccination has been shown to increase the numbers of liveborn pigs per litter and to decrease farrowing intervals (52). Breeding herd vaccination has also been shown to decrease the incidence of periparturient vulval discharge (52). Consequently, vaccination against *E. rhusiopathiae* at every breeding cycle is standard in most pig breeding herds.

Protective immunity after vaccination is generally thought to range between 4 and 6 months. Thus, pig breeding herds are typically re-vaccinated in every cycle or at least twice a year. It has been suggested that in-feed antibiotics could negatively impact vaccine responses, especially when using attenuated erysipelas vaccines (108). However, a study using selected antibiotics could not find major differences between treatment groups, and vaccinated pigs were protected from pathogenic challenge (108). In a similar study by a different group, using 105 8-week old pigs, it was found that vaccination of pigs against erysipelas in the presence of antibiotics may result in a decrease (ceftiofur, doxycycline, tiamulin) or enhancement (amoxillin, tulathromycin) in the production of specific antibodies against *Erysipelothrix* spp. (106). In contrast, the use of ginseng as a co-adjuvant has been shown to improve the antibody response of vaccination (109). Ginseng contains immunomodulators named ginsenosides, which in pigs enhance the antibody response to viral and bacterial antigens found in vaccines. Two different erysipelas bacterins were tested and the addition of ginseng improved the less immunogenic vaccine so that it became more immunogenic compared to the vaccine without ginseng. The authors concluded that the use of ginseng as a co-adjuvant provides a simple, safe and inexpensive alternative for improving the potency of aluminum hydroxide adjuvanted vaccines (109). Vaccination against other pathogens has not been shown to affect the efficacy of erysipelas vaccines. When pigs were vaccinated with an attenuated porcine reproductive and respiratory syndrome virus (PRRSV) vaccine and later with an attenuated erysipelas vaccine followed by erysipelas challenge, a negative impact of PRRSV vaccine on the erysipelas vaccination was ruled out (110). Besides their usage in farmed animals, *Erysipelothrix* spp. vaccines are occasionally used off-label in other species, including marine mammals, with encouraging results (111, 112). Marine mammals are highly susceptible to *Erysipelothrix* spp. infections, the disease course is almost always acute and is commonly fatal (113). Protection against *Erysipelothrix* spp. isolates recovered from dolphins has been demonstrated in mice vaccinated with a commercial pig bacterin (114). The off-label use of an attenuated swine vaccine in laying hens (35) or turkeys (77) has also been reported.

## Autogenous Vaccines

For veterinary purposes, autogenous vaccines refer to “any immunological veterinary medicinal products manufactured (by a qualified person) for the purpose of producing active immunity from pathogenic organisms obtained from an animal or animals from the same herd that have been inactivated and used for the treatment of this animal or of animals from this herd” (Article L 5141-2 of the Public Health code; https://www.biovac.ceva.com/en/Autogenous-vaccines/Autogenous-Vaccines accessed 09-Mar-2020). Autogenous vaccines are popular in the veterinary field, especially in pigs and poultry. They are commonly used in pig or poultry erysipelas outbreaks, usually when there is a perceived lack of commercial vaccine efficacy or when there are no suitable products on the local market. Such vaccines, as outlined above, are limited in their usage to the farm the microorganism originated from. Advantages include the availability of farm-specific isolates to protect the affected herd, while the main disadvantage is the absence of vaccine validation work including dose determination, adjuvant selection and antigen load, which are instead based on estimates from other vaccines or experience. In addition, autogenous vaccines may be accidentally contaminated with other pathogens. In 2004, autogenous vaccines accounted for 15–20% of the US pig market (115). Specifically for erysipelas, of all licensed erysipelas vaccines in the USA during 2019 62.2% were attenuated, 28.6% were inactivated and 9.2% were autogenous (Source: USDA, Dr Paul Hauer).

## Cross-Protection Among *Erysipelothrix* spp. Isolates

Research trials investigating cross-protection among *Erysipelothrix* spp. isolates are still limited due to the large number of strains corresponding to most serovars and a lack of *in vitro* models that could be used. Vaccine trials have been conducted in pigs, mice and turkeys. For each trial appropriate positive and negative controls need to be included, and with 3R (replacement, reduction, refinement) regulations it is not always feasible to test more than one isolate per serovar and more than the most commonly occurring serovars. A summary of published trials (77, 105, 116–123) is provided in Table 2.

**Table 2:** Cross-protection trials for *Erysipelothrix* spp., including host species the trial was conducted in (mouse, pig, or turkey), and serovars and Spa details in the vaccines and challenge isolates.

**Vaccine Type**	**Isolate**	**Serovar**	**Spa type**	**Route^a^**	**Dose**	**Species**	**Number of animals per serovar (Total)**	**Number of isolates within a serovar**	**Serovar used for the challenge^b^**		**References**
									***Protected***	***Not protected (% mortality)***	
Inactivated	AN-4, SE-9, CN3342, CN3461	2	A	SC	1×	Mouse	30-60 (180)		1,2,4,11	9,10 (57–77%)	(117)
					2×	Pig	3-6 (33)		1,2,4,11	9,10 (70–83%)	
Inactivated	AN-4, SE-9, CN3342, CN3461	2	A	SC	1×	Mouse	10 (1200)	10 serovar 1, 2, 4, 9, 10 and 11 isolates	1,2	4,9,10,11 (9–51%)	(105)
					2×	Pig	8 (64)		1,2	9,10 (37.5–100%)	
Inactivated	Kyoto	2	A	IM	2×	Pig	2 (6)		1a,2		(124)
Attenuated	Koganei 65-0.15	1a	A	SC	1×	Pig	1 (1)		1a		
Attenuated	Koganei 65-0.15	2^b^	A	SC	1×	Mouse	10 (790)		1a,1b,2,3,5,6,7,8,11, 12,15,16,18,19,21,N	10,14,20,22 (20–30%)	(116)
				ID	1×	Pig	2 (78)		1a,1b,2,5,8,11,12,18, 19,21	9,10 (100%)	
Attenuated	Koganei 65-0.15	2^b^	A	SC	1×	Mouse	10 (200)		4,6,7,8,9,10,15,16,N	20 (30%)	(121)
						Pig	2 (40)		4,6,7,9,15,16,N	8,10,20 (50%)	
Attenuated	Koganei 65-0.15	2^b^	A	SC	1×	Mouse	10 (400)	Two serovar 8 isolates	1b,2,8,N	1a,4,5,6,7,8,11,12, 15,16,21 (20–50%) 9,10,18,19,20 (60–100%)	(123)
						Pig	2 (40)	Two serovar 8 isolates	1a,1b,2,4,5,6,7,8,9,10, 11,12,15,16,18,19,21,N	20 (50%)	
Attenuated	EW-2	1a	A	Oral	1 × 2×	Turkey	10–13 (82) 10–13 (82)			1a (15–54%) 1a (10–70%)	(77)
Inactivated	Ersipelin, Fort Dodge Lab	Unknown		SC	1×		4-8 (64)		1a		
Subunit	Not applicable		A	SC	2×	Mouse Pig	Not indicated 2(10)		1a 1a,2		(122)
Subunit			A	SC	2×	Mouse	40		1a, 2	6,18 (30–50%)	(120)
	Not applicable		B		2×		40		6	1a,2,18 (40–50%)	
			C		2×		40		6,18	1a,2 (10%)	
Inactivated	SE-9	2	A	SC	1×	Mouse	10–12 (10)		1a,2,N	2/15^e^,N,5,6 (30–100%)	(118)
Subunit			C^c^	SC	2×	Mouse	10 (200)		1a,18,19		(119)
			C^d^	IM	2×	Pig	3–6 (9)		1a		

a *Administration route: SC, subcutaneous; IM, intramuscular; ID, intradermal*.

b *This strain is indicated as serovar 2 in these publications*.

c *Full length or N terminal-half region*.

d *N terminal-half region*.

e *Isolate cross-reacted with antisera against serovars 2 and 15*.

## Protective Antigens

Already in 1970 a protective antigen was described from an *E. rhusiopathiae* culture (86). In 1990, a 64 to 66-kDa protein in cell wall extracts was shown to be protective (125). However, the genetic fragment, which was cloned in a lambda phage vector and encoded the protein, had not been sequenced at the time. In 1998, the major protective antigen of *E. rhusiopathiae*, designated as SpaA, was identified and cloned in *Escherichia coli* and SpaA has since been fully characterized (89, 90). The SpaA protein, whose presence is thought to be highly conserved across *E. rhusiopathiae* isolates, is not present in *E. tonsillarum* (30, 31, 37, 120, 126). In 2007, the *Erysipelothrix* spp. Spa protein was classified into three types, designated as SpaA, SpaB, and SpaC (120). The amino acid sequence similarity within each Spa type was found to be high (96–99%) but among different Spa types it was rather low (~60%). The greatest diversity in Spa proteins was found in the N-terminal half of the molecule (50–57% similarity) (120). Most identified *Erysipelothrix* spp. isolates from farmed animals contain SpaA. The protective domain of SpaA, cloned from isolate Fujisawa (serovar 1a), was demonstrated to be between amino acid residues 12 to 195 of the protein based on a mouse challenge study (90). In recent years *Erysipelothrix* spp. isolates containing SpaC have been associated with increased fish mortality (16) in the USA and very recently SpaC has also been identified in *Erysipelothrix* sp. 2 isolates from turkeys with increased mortality in Brazil (127).

With additional molecular tools becoming available and also affordable, several research groups have investigated *Erysipelothrix* spp. for the presence of different protective antigens and many of those have also been further characterized. Protection against *Erysipelothrix* spp. is generally mediated by antibodies against antigens located at its surface and certain antigens present in the culture supernatant (66). The first genomic sequence of *E. rhusiopathiae* became available in 2011 and indicated presence of a complete set of peptidoglycan biosynthesis genes, two-component regulatory systems, and various cell wall-associated virulence factors, including a capsule and adhesins (1). The capsule above all plays an important role in immune evasion; however, the capsule of *E. rhusiopathiae* is poorly immunogenic and the antibodies against the capsular antigen are not protective (128, 129).

Other *E. rhusiopathiae* protective antigens reported so far include RspA (*rhusiopathiae s*urface *p*rotein A) (130), CbpB (*c*holine-*b*inding *p*rotein) (131, 132), and GAPDH (*g*lycer*a*ldehyde-3-*p*hosphate *d*e*h*ydrogenase) (133). These protective antigens play important roles in biofilm formation (RspA) (130) and adhesion to porcine endothelial cells (SpaA and GAPDH) (133, 134), and to extracellular matrix proteins, including fibronectin (RspA and GAPDH) (130, 133), collagens (RspA) (130), and plasminogen (GAPDH) (133). All of these protective antigens are surface exposed and the protective roles of these proteins are conferred by opsonophagocytic killing by macrophages. While all the protective antigens that have been proposed appear widespread within *E. rhusiopathiae* (135), it is unknown whether differences in these antigens result in varying degrees of cross-protection.

## Immunological Basis or Correlates of Protection for Existing and Novel Vaccine Candidates

By far the best way to assess vaccine efficacy is to do a live pathogen challenge. However, this is not always possible due to cost, animal species assessed (pathogen challenges would be impossible in cetaceans), the high numbers of *Erysipelothrix* spp. isolates circulating in animals, animal welfare reasons, and lack of availability of appropriate research facilities or relevant permits or both. Therefore, when assessing vaccines and vaccine efficacy one of the first correlates of immunity measured is antibody levels in vaccinated animals, most commonly IgG but also IgM, to confirm that a B-cell response took place. Several assays including agglutination assays, indirect immunofluorescence assays (IFA), ELISAs, and fluorescent microbead immunoassays (FMIAs) have been developed for *E. rhusiopathiae* (38). In experimentally vaccinated pigs, IgM and IgG responses can be demonstrated between 7 and 21 days post vaccination (136–140). A study using conventional pigs found that vaccination of pigs with a commercial inactivated vaccine in the presence of antibiotics may result in a decrease (ceftiofur, doxycycline, tiamulin) or enhancement (amoxicillin, tulathromycin) in the production of specific antibodies as measured by a commercially available *E. rhusiopathiae* ELISA (106). During assessment of a large vaccination program in various marine mammals by a modified FMIA, a mean 311-fold increase in the IgG antibody index was detected 14 days after the first booster vaccination (112). In that study serum IgG antibody titers were influenced by the number of vaccinations received but not by age, sex, history of natural infection, adverse vaccine reaction, vaccination interval or time since last vaccination (112). When investigating vaccine responses in endangered species, it may be necessary to develop a novel assay. An ELISA was developed to assess IgY antibodies in kakapos before and after vaccination (141). The results indicated a possible transfer of maternal IgY molecules to fledglings via the yolk. Evidence that vaccination increased the kakapo population's mean anti-*E. rhusiopathiae* IgY levels was lacking. The authors concluded that vaccination may only raise the IgY levels of birds with relatively low pre-vaccination IgY levels (141). In New Zealand, an existing swine erysipelas ELISA was modified to assess swine erysipelas vaccination in layer birds (142). The domestic poultry were vaccinated twice with an inactivated pig vaccine at low (2 ml) or high (4 ml) dose on days 0 and 21. Optical density readings were higher on days 21, 42, and 63 than day 0 in both groups suggesting that vaccination using either dose induced detectable levels of antibody. The authors concluded that the ELISA will be useful for monitoring responses to vaccination in future (142).

Another possibility to assess whether an animal has been vaccinated and responded appropriately to the vaccination is to measure cell mediated immunity (CMI). This is typically done by enzyme-linked immune absorbent spot (ELIspot) assays, lymphocyte proliferation assays (140, 143), or by assessing presence and amount of cytokines by molecular methods (144). These assays are labor intensive, rely on fresh blood samples that are processed within hours of collection, and are currently only used in selected research laboratories. A study in cetaceans vaccinated off-label with a commercial swine erysipelas vaccine found a vaccine-induced interferon γ response consistent with a T helper 1 (T_H_1) response which was correlated with lack of clinical erysipelas in that group (144). Furthermore, the same study also compared 6 and 12 month booster vaccinations. While a superior memory response was found in the group re-vaccinated 6 months later, anamnestic responses were only identified in the group re-vaccinated every 12 months (144).

Some studies compared or correlated both humoral immunity and CMI. Under field conditions, most growing pigs will have maternally derived antibodies (MDAs) due to regular breeding herd vaccination, and vaccine efficacy may be difficult to assess using serological assays unless paired serum samples (pre-vaccination and post-vaccination) are available. Previously it was determined that presence of MDAs may interfere with vaccination and subsequent protection (138, 139). The effect of MDAs on the immune response to an oral live *E. rhusiopathiae* vaccine given at 6, 8, or 10 weeks of age was investigated in conventional pigs (140). A clear seroconversion was only detected in pigs vaccinated at 8 or 10 weeks of age. The CMI response was assessed by a lymphocyte proliferation assay and the investigators found a response in 25% of piglets vaccinated at 6 weeks of age and in 100% of piglets vaccinated at 8 or 10 weeks of age (140). In an Australian study, isolates derived from six herds affected by erysipelas vaccine breakdowns were utilized and responses to commercial and experimental bacterins were assessed (143). The investigators found significantly different humoral and CMI responses (determined by a lymphoproliferation assay) among treatments. While a similar antibody response against a serovar 2 lysate was induced by all vaccines, only those providing significant protection against serovar 1 produced significantly elevated antibodies against the serovar 1 lysate. Vaccination in general significantly reduced CMI responses to the mitogens concanavalin A and phytohaemagglutinin. The results were confirmed in an experimental pig challenge system. The most effective vaccine response was associated with the highest mean serovar 1 antibody response and the highest CMI response to serovar 2 (143).

## Evidence of Vaccine Failures, Vaccine Mismatches, and Inability to Provide Cross-Protection

Erysipelas vaccine failures are not always reported and case reports are rare. Over the last few decades, *E. rhusiopathiae* outbreaks were commonly observed whenever established erysipelas vaccination programs were discontinued in an effort to reduce production costs. Failure to properly vaccinated pigs has been documented (145). This was likely also the case for the last large erysipelas outbreak in the USA during 2000–2001 (32). However, in recent years evidence has grown to support that current vaccines may not always be effective.

In Australia between 1995 and 1998, vaccine failures were reported in four different states (30). The majority of the outbreaks were due to serovar 2 isolates but further characterization with available methods at that time could not identify any single clonal population responsible for the outbreaks (30). As stated in the previous section, challenge studies including CMI and antibody analysis indicated a lack of protection against serovar 1 isolates (143).

More recently, during 2015-2016, a continuous grow-finish farm located in the UK experienced recurring outbreaks of erysipelas in 18–22 week-old pigs (43). Clinically, there was delayed average daily gain, a high incidence of lameness and ear discolorations, with an average morbidity rate of 8–12%. *E. rhusiopathiae* serovar 15 was isolated from lesions on three different occasions. The source breeding herd was routinely vaccinated with a commercial serovar 2 bacterin which was switched to another bacterin during the investigation without much improvement. Cross-sectional serological assessment of the outbreak farm revealed a lack of anti-*Erysipelothrix* SpaA antibodies up to ~14 weeks of age. As treatments were not effective, the herd was eventually depopulated (43).

The first documented outbreak of erysipelas in chickens dates back to 1958 (146). Outbreaks of erysipelas in layers have been reported since (35). Commonly the layers are not being vaccinated at the time of the erysipelas outbreak, and subsequent introduction of vaccination programs resolves these situations (35). In Europe, where poultry-dominant countries have relied on erysipelas vaccines for many years, increasing outbreaks in chickens are being observed in various countries including Denmark (48), Germany (46), Sweden (147), the United Kingdom, and the Netherlands (TO, unpublished observations). Erysipelas outbreaks were also described in commercial geese in Poland (44). Affected flocks are often outdoor and organic operations and they are almost always layers. A Swedish study previously confirmed an association of erysipelas in laying hens with housing system (148). Specifically, the risk for an outbreak was higher in free-range systems than in indoor litter-based systems, and lowest for flocks housed in cages (148). Vaccines currently used in European poultry are inactivated and based on serovar 2. Interestingly, the dominant serovars from chicken flocks with outbreaks include 1b and 5 (TO, unpublished observation).

In a more molecular approach to assessing the relationship between field and vaccine strains, the genomic and immunogenic protein diversity of *E. rhusiopathiae* isolates from pigs obtained between 1987 and 2014 was analyzed and compared to the currently predominant vaccine strain in the UK, an inactivated serovar 2 vaccine (149). While all British pig isolates had one amino acid difference in the 385-amino acid immunoprotective domain of the SpaA protein compared to the vaccine strain, *in silico* structural protein analyses suggested that this difference is unlikely to compromise vaccine protection. The authors hypothesized that the observed sequence variants in surface proteins could be responsible for differences in the efficacy of the immune response (149). This work was solely based on serovars 1a, 1b and 2 from pigs and should perhaps be expanded to other commonly found serovars and species affected including chickens and serovar 5.

## Future Directions in Vaccine Development

Today, commercial erysipelas vaccines, either attenuated or inactivated, are based on a small number of *E. rhusiopathiae* strains isolated several decades ago. Regardless of the vaccine type, *E. rhusiopathiae* vaccines are generally considered effective in preventing erysipelas. However, if vaccines are not fully effective or retain residual virulence, chronic forms of the disease, which tend to follow the acute form, may develop. In Australia, outbreaks due to vaccine breakdowns of multivalent and bivalent vaccines have been reported (30). Recently, it was reported that the acriflavine-resistant *E. rhusiopathiae* vaccine (Koganei 65-0.15 strain), developed from serial passage in the presence of acriflavine mutagen, may have reverted to a virulent strain *in vivo* and is now associated with clinical disease in Japan (150). Thus, erysipelas vaccines developed using empirical approaches may not represent the best vaccines and require improvement.

Many vaccine platforms have become available over the last decades, including purified microbial components, subunit vaccines based on polysaccharide-carrier protein conjugates or recombinant proteins, DNA vaccines, nanovaccines, and others. New approaches and strategies for vaccine development against swine erysipelas have been achieved and have reached the pre-clinical stage. These include subunit vaccine candidates (Table 3) and attenuated vaccine candidates (Table 4). Microcrystalline cellulose (Avicel PH-101) as a delivery carrier of recombinant protein-based antigen has been assessed by fusing SpaA to a cellulose-binding domain from the fungus *Trichoderma harzianum* endoglucanase II through a S3N10 peptide (153). The fusion protein was expressed in *E. coli* and subsequently bound to Avicel PH-101. The vaccine was tested in the mouse model and provided 100% protection in mice against challenge with a serovar 15 isolate (153). While this particular vaccine candidate appears promising it needs to be verified using pigs.

**Table 3:** Novel approaches and strategies for subunit erysipelas vaccines.

**Vaccine type**	**Antigen^a^**	**Expression vector**	**Adjuvant**	**Challenge serovar**	**Species**	**Number (route)^b^**	**Survival**	**References**
Subunit	SpaA	*E. coli*	Whole *E. coli* cells	2 (Tama-96)	Mice	10 (IP)	100%	(89)
Subunit	SpaA	*E. coli*		1a (Fujisawa)	Mice	5 (SC)	100%	(90)
						5 (IP)	100%	
Subunit	SpaA	*E. coli*	Freund's adjuvant	1a (Fujisawa)	Pigs	4 (IM)	100%	(122)
				2b (82–875)		2 (IM)	100%	
Subunit	SpaA	*Bacillus brevis*	*E. coli* heat-labile enterotoxin	1a (Fujisawa)	Pigs	6 (IN)	100%	(151)
Subunit	CbpB	*E. coli*	Freund's adjuvant	1a (Fujisawa)	Mice	10 (IM)	80%	(131)
					Pigs	7 (IM)	86%	
Subunit	GAPDH	*E. coli*	Freund's adjuvant	1a (SE38)	Mice (C57BL/6)	10 (IP)	100%	(152)
	HP0728					10 (IP)	0%	
	HP1472					10 (IP)	0%	
	CbpB-N					10 (IP)	50%	
	SpaA					10 (IP)	100%	
	None (PBS)					10 (IP)	0%	
	GAPDH	*E. coli*	Montanide ISA 206	1a (SE38)	Pigs	5 (SC)	80%	
	SpaA					5 (SC)	100%	
	None (PBS)					5 (SC)	0%	
Subunit	Soluble CBD-SpaA	*E. coli*	None	15	Mice	8 (SC)	75%	(153)
	Coated CBD-SpaA		Avicel^c^			8 (SC)	100%	
	ERT2T-A containing whole bacterin		None			8 (SC)	62.5%	
	None (PBS)		None			8 (SC)	0%	

a *SpaA, surface protective antigen A; GAPDH, glyceraldehyde-3-phosphate dehydrogenase; CbpB, choline-binding protein B; PBS, Phosphate-buffered saline; CBD, cellulose-binding domain*.

b *Administration route: IP, intraperitoneal; SC, subcutaneous; IM, intramuscular; IN, intranasal*.

c *Micro-crystalline cellulose*.

**Table 4:** Novel approaches and strategies for attenuated erysipelas vaccines.

**Vaccine type**	***E. rhusiopathiae* strain**	**Specifics**	**Dose (route)^a^**	**Challenge serovar**	**Species**	**No. of animals used**	**Survival**	**References**
Attenuated (vectored)	YS-1	R1^b^ and R2 regions of the P97 adhesin of *M. hyopneumoniae* strain E-1 fused with SpaA	2 (IN) 1 (IN)	1a	Pigs	3 3	100% 0%	(154)
	Koganei 65-0.15		7 (oral)	1a	Pigs	8	100%	(155)
Attenuated	Δ432	651 mutants were screened in mice to determine attenuation and protective immunity	2 (oral) 1 (oral)	1a	Pigs	10 10	100% 90%	(150)

a *Administration route: IN, intranasal*.

b *R1 and R2 are repeat regions*.

In the swine industry, where cost effective vaccines are strongly required, one of the most challenging strategies is the development of vectored vaccines which can prevent different diseases simultaneously and are inexpensive to produce. Due to the ability of *E. rhusiopathiae* to effectively induce both humoral and cell-mediated immune responses, attenuated *E. rhusiopathiae* isolates have been used for this purpose. It was shown that attenuated *E. rhusiopathiae* vaccines expressing the recombinant protein of the P97 adhesin of *Mycoplasma hyopneumoniae* could induce protective immunity against a lethal *E. rhusiopathiae* challenge and also reduce mycoplasmal lesions following experimental infection with a virulent *M. hyopneumoniae* when administered intranasally (154) and orally (155). It was also found that a single intradermal injection with a needle-free injector of a vectored vaccine was effective against the mycoplasmal infection (YS, unpublished observation). Thus, attenuated *E. rhusiopathiae* isolates may be used as platform vectors for live delivery of heterologous antigens through the oral and parenteral routes. Very recently, based on genome-wide screening for *E. rhusiopathiae* virulence-related genes, an *E. rhusiopathiae* mutant deficient in a *tagF* homolog (encoding a putative CDP-glycerol glycerophosphotransferase) proved to be a safe and effective vaccine candidate that can be administered via the oral and subcutaneous routes (150). The *tagF* mutant may be the best choice for the development of vectored vaccines.

Another innovative strategy to protect against *Erysipelothrix* spp. is utilization of the protective epitope of SpaA protein. It has been shown that SpaA protein is a major protective antigen and the antibodies against the N-terminal one third of the protein play an important role in protection (90). If the protective epitope within this region is identified, the epitope sequences can be included into subunit or DNA vaccines against other pathogens. With the expansion of next generation sequencing, several draft genome sequences of *Erysipelothrix* spp. isolates have become publicly available (37). Capitalizing on this genome sequence data, bioinformatics approaches may enable identification of novel protective epitopes and/or antigens for possible future vaccine candidates (156).

Changes in vaccine administration may also need to be considered. With more and more outdoor poultry affected by erysipelas, parenteral vaccination may be less suitable, as outdoor poultry can be difficult to catch and vaccinate, especially in larger flocks. Alternatives include *in ovo* vaccination, nasal vaccination via dust or drop, oral vaccination via the feed, or vaccination via the drinking water similar to pigs or via spray using atomizers. While the ease of vaccination is important, cost is even more important in poultry and needs to be addressed with any new vaccine administration route.

A final necessary consideration into the vaccine efficacy of *Erysipelothrix* spp. is the compatibility between vaccine and field isolates. Current vaccines are mostly based on *E. rhusiopathiae* strains isolated several decades ago. Whether these remain effective in providing protection against globally circulating isolates is an important area for investigation. With increasing whole genome sequences of *E. rhusiopathiae* from various sources available within the public domain, comparative genomic studies are expected to provide valuable insights into these questions.

Thus, exciting studies have been published on novel *E. rhusiopathiae* vaccine concepts and more research will likely become available in the near future. The importance of testing novel vaccine candidates on a regular basis, side-by-side with trials performed by independent and unbiased institutions to confirm promising candidates and discard the others at a very early stage, should be underscored.

## Summary and Conclusions

The history of erysipelas is probably as old as pig domestication, and, for many centuries, this devastating disease resulted in high morbidity and mortality in livestock species. Since the first isolation of *Erysipelothrix* spp. in 1876 and its link to swine erysipelas in 1882, several important milestones have led to the development of safe and effective vaccines. These are nowadays widely used in farmed pigs, poultry and lambs, as well as in highly susceptible individuals such as marine mammals in commercial aquarium settings. However, a major persistent knowledge gap is the limited to non-existent understanding of the factor(s) that confer protective immunity (i.e., whether a similar serovar, genotype, Spa type or other protective antigen is needed to confer cross-protection, or any combination of these). For example, if an erysipelas outbreak in a vaccinated population occurs, it is currently unknown if serovar is of any importance for cross-protection. Yet, in the field, serotyping of erysipelas isolates in outbreak scenarios is commonly requested by practitioners and results are used to make vaccine decisions, i.e., whether to switch from a serovar 1 vaccine to a serovar 2 or vice versa or to have an autogenous vaccine produced. As outlined, Spa type could also be of importance. However, most erysipelas isolates from farmed animals contain SpaA so excellent cross-protection should be expected. This is based on the assumption that *spaA* genes are similar across *Erysipelothrix* spp., which is presently poorly characterized. With vaccine failures increasingly reported in pigs and poultry, and erysipelas also emerging in various wild animal reservoirs, investigations into factors associated with protective immunity are warranted, ultimately leading to novel, updated vaccine candidates for improved protection against erysipelas.

## Author Contributions

TO drafted the original manuscript. YS and TF corrected the draft and added relevant information. All authors read and approved the final manuscript version.

### Conflict of Interest

The authors declare that the research was conducted in the absence of any commercial or financial relationships that could be construed as a potential conflict of interest.
